# Xanthogranulomatous prostatitis: a mimic of carcinoma of prostate

**DOI:** 10.1186/1477-7819-4-30

**Published:** 2006-06-05

**Authors:** Muhammad Rafique, Nausheen Yaqoob

**Affiliations:** 1 Department of Urology, Nishtar Medical College, Multan, Pakistan; 2 Department of pathology, Agha Khan University Hospital, Karachi, Pakistan; 3 5, Altaf Town, Tariq Road. Multan, Pakistan

## Abstract

**Background:**

Xanthogranulomatous prostatitis is an unusual benign inflammatory process of prostate. Clinically it mimics prostatic carcinoma, requiring pathological examination for diagnosis.

**Case presentation:**

A 60-year-old patient presented with 6 months history of increasing difficulty in micturition. On digital rectal examination prostate was hard and nodular and estimated weight was 50-gram. His serum prostate specific antigen (PSA) was 150 ng/ml. Clinically a locally advanced carcinoma of prostate was suspected. In view of severe obstructive urinary symptoms and significant post-micturition residual urine, transurethral resection of prostate was carried out. Histopathological examination of resected prostatic tissue revealed xanthogranulomatous prostatitis with no evidence of malignancy. Patient remains symptom free at 16 months follow-up and serum PSA has decreased to 6 ng/ml.

**Conclusion:**

Xanthogranulomatous prostatitis is a benign inflammatory disorder of prostate that can clinically and even biochemically mimic prostatic carcinoma. A high degree of suspecion and close co-operation with pathologist is necessary for the diagnosis of xanthogranulomatous prostatitis.

## Background

A variety of the granulomatous lesions of the prostate have been described with varied etiology and pathogenesis [1]. Xanthogranulomatous prostatitis is one such rare benign inflammatory lesion of prostate that can clinically mimic prostatic carcinoma. Sometimes condition may be mistaken for high-grade prostatic carcinoma [2].

Herein we report a patient who had xanthogranulomatous prostatitis but initially on clinical and biochemical grounds, he was mistakenly diagnosed to be case of locally advanced prostatic carcinoma.

## Case presentation

A 60-years-old man presented with 6 months history of increasing difficulty in micturition, He had hesitancy, weak flow, intermittency and increased urinary frequency. His American Urinary Association (AUA) symptom score was 24, consistent with severe prostatic symptoms. He had no significant past medical history and was non-diabetic. His general physical examination was normal. On digital rectal examination prostate was enlarged with approximate weight of 50 grams. It was non tender and felt hard and nodular. A provisional diagnosis of locally advanced prostatic carcinoma was made. His renal function tests and complete blood counts were normal. Erythrocyte sedimentation rate (ESR) was elevated at 55 mm at 1 hour. Routine urinalysis revealed 8–10 WBC's/hpf but urine culture was negative. PSA was significantly elevated at 150 ng/ml (normal 0–4 ng/ml). At transabdominal ultrasonography he had normal upper renal tract. Bladder was thick walled and prostate was 5 × 5 × 4 Cm in size and was rather uniformly hypoechoic. Post-micturition residual urine was 160 ml. His chest, plain x-ray KUB and radioisotope bone scan was normal. Urine flow rate at flowmetry was 12 ml/sec.

In view of the troublesome obstructive urinary symptoms, significant residual urine, needle biopsies were not performed and he underwent cystoscopy and transurethral resection of prostate (TURP). At cystoscopy, prostatic urethra was inflamed. Prostate was quite occlusive with irregular intra-vesical protrusion. Bladder was trabeculated and was generally congested. TURP was carried out, 54 gm tissue was resected and it amounted to near complete resection. During resection, prostatic chips were rather yellowish but no abscess cavities or calculi were encountered. His postoperative recovery was uneventful.

**Figure 1:**
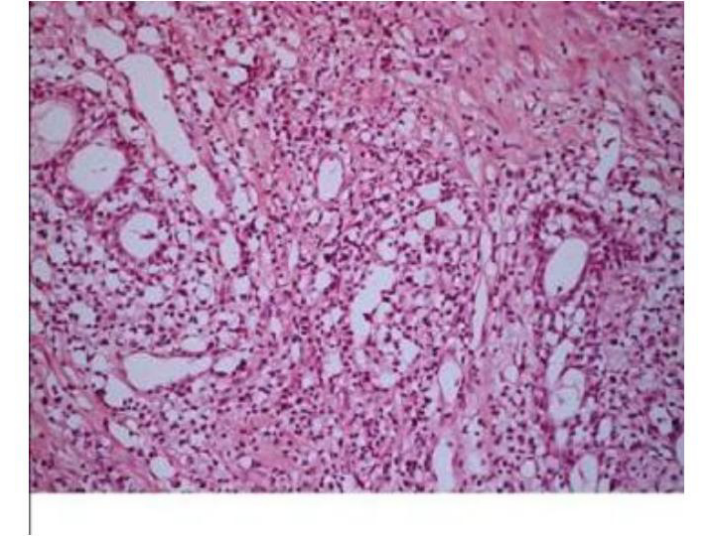
Photomicrograph showing intense infilteration of prostate with lymphocyte, plasma cells and xanthoma cells (marked with arrow). H & E × 200.

Histopathology of the rescected tissue revealed dense xanthogranulomatous inflammation (Figure 1) mixed with eosinophils and foci of calcification. Benign prostatic glands were identified and no evidence of malignancy was noted. At 16 months follow-up patient remains symptom free. His serum PSA has decreased to 6 ng/ml.

## Discussion

A variety of granulomatous lesions of prostate have been described. Excluding the few cases in which the etiologic agent can be identified, the classification of the granulomatous lesions within the prostate remains controversial [1]. Infective agents that have been implicated in the specific granulomatous prostatitis include various fungi, *Treponema pallidum *and most commonly *Mycobacterium tuberculosis*. The most commonly diagnosed granulomatous lesion within the prostates has been of nonspecific granulomatous prostatitis [3,4].

The etiology and pathogenesis of this morphologically distinct lesion remains unknown. It is thought to represent a reaction to inflammatory products and altered prostatic secretions released from obstructed ducts [5]. Recently one study has linked this condition to an autoimmune disease [6]. The typical lesion in granulomatous prostatitis consists of a large nodular infiltrate of epitheliod histiocytes, lymphocytes and plasma cells occupying many prostatic lobules.

The distinctive feature of xanthogranulomatous prostatitis is the presence of large number of "foamy macrophages" (histiocytes) in the inflammatory cell infiltrate. Using an immuno-histological techniques, "T" lymphocytes are in close association with damaged epithelium while "B" lymphocytes occur in more peripheral location or form follicular structures [7]. A xanthogranulomatous pattern or prominence of epithelioid histiocytes sometimes bears a resemblance to high-grade prostatic carcinoma [8] and immunohistochemical panel has been proposed that can reliably distinguish between these two conditions [9]. However, on rare occasions granulomatous prostatitis and prostatic carcinoma may coexist [10].

Xanrthogranulomatous inflammation is well known in the kidney and gallbladder but prostate is rare site for this lesion. Less than 10 cases have been reported in the literature.

Average age at the time of diagnosis is early sixties, with a wide range from twenties to the very elderly. Clinically the symptoms are those of either urinary obstruction or a severe lower urinary tract infection [5]. On digital rectal examination, it is difficult to distinguish from prostatic carcinoma [11] as the prostate feels hard and nodular. In addition, this condition can cause elevation in serum PSA level. In one study, serum PSA ranged from less than 0.5 ng/ml to 114 ng/ml (mean 12.7 ng/ml) [4]. This increase of PSA level is usually transient [12]. In the present case serum PSA was markedly elevated at 150 ng/ml and this high level of PSA has not been reported in the literature previously. In spite of the very high serum PSA his bone scan was within normal limits. After transurethral resection of prostate, serum PSA level decreased to 6 ng/ml. On transrectal ultrasonography (TRUS) and magnetic resonance imaging (MRI) there is no pattern that allows a specific diagnosis of granulomatous prostatitis or differentiate it from prostatic carcinoma [13]. Hence, the diagnosis of xanthogranulomatous prostatitis is made on histological examination of prostate.

## Conclusion

Xanthogranulomatous prostatitis is uncommon and may simulate prostatic carcinoma both clinically and microscopically. There are no specific radiological features. Knowledge of this condition and close co-operation with pathologist is necessary for the diagnosis of xanthogranulomatous prostatitis.

## Competing interests

The author(s) declare that they have no competing interests.

## Authors' contributions

**MR**: Preparation of manuscript, **NY**: Histopathological examination of submitted prostatic tissue and preparation of microphotographs. Both authors read and approved the final manuscript.
